# Envelope-Fusion-Syncytium Formation in *Microplitis bicoloratus bracovirus* Maturation

**DOI:** 10.3390/v14102183

**Published:** 2022-10-02

**Authors:** Ming-Wu Dai, Kai-Jun Luo

**Affiliations:** 1 School of Life Sciences, Yunnan University, Kunming 650500, China; 2 Key Laboratory of the University in Yunnan Province for International Cooperation in Intercellular Communications and Regulations, Yunnan University, Kunming 650500, China

**Keywords:** electron microscopy, fused envelope, syncytium formation, *Microplitis bicoloratus bracovirus*, viral maturation, phagocytic calyx epithelial cells

## Abstract

The viral envelope is essential for virus maturation. Virus-mediated syncytium formations are induced by viral envelope proteins that cause membrane fusion of the infected cells. *Polydnaviridae* (Polydnavirus) are enveloped viruses with multiple nucleocapsids, and virions mature in symbiotic parasitoid wasp ovaries. However, the mechanism governing the envelope packaging of multiple nucleocapsids remains unclear. In this study, we used transmission electron microscopy to examine the process whereby multiple nucleocapsids of *Microplitis bicoloratus bracovirus* are packaged into an envelope and observed envelope-fusion-syncytium formation in symbiotic wasp calyx cells during virus maturation. The virus maturation process in calyx cells comprised four stages: pre-virogenic stroma, virogenic stroma, assembly, and fusion. Each virus contained a single envelope with one nucleocapsid in the assembly stage; multiple envelopes then fused to form a viral envelope with multiple nucleocapsids (i.e., the envelope-fusion-syncytium) around the envelope fusion core in the fusion stage. The envelope-fusion-syncytium then stabilized the virions that were released into the lumen of the ovary across the calyx epithelial layer. The phagocytic calyx epithelial cells on the border of the calyx and ovary lumen cleared the majority of non-enveloped nucleocapsids. In contrast, non-phagocytic calyx epithelial cells with microvilli and a cuticular line between the ovary wall and the lumen remained intact in the ovary lumen. These results indicate that envelope-fusion-syncytium formation is important for packaging multiple nucleocapsids in bracovirus maturation.

## 1. Introduction

*Polydnaviridae* (Polydnavirus) are enveloped multiple-nucleocapsid viruses with a symbiotic relationship with parasitoid wasps. Based on two symbiotic parasitoid wasp families, Braconidae and Ichneumonidae, polydnaviruses (PDVs) are divided into two genera: *Bracovirus* (BV) and *Ichnovirus* (IV), respectively [1]. BV is surrounded by one envelope, which is formed de novo in the nucleus, and is released via lysis of the nuclear and plasma membranes. In contrast, IV is covered by two envelopes, with its inner envelope formed de novo in the nucleus and its outer envelope acquired from the plasma membrane via plasma membrane budding [2]. Further, the mature virions contain multiple nucleocapsids [3,4,5].

Virus-mediated syncytium formation is induced by viral envelope proteins, which cause membrane fusion of the infected cells [6,7,8,9,10]. However, viral envelope fusion has not been reported previously. Multiple nucleocapsids are packaged in the mature *Microplitis bicoloratus bracovirus* (MbBV) virion envelope in the calyx cells of wasp ovaries. However, the underlying mechanism whereby multiple nucleocapsids are packaged into the envelope remains unclear. The links between envelope proteins that mediate syncytium formation in virus-infected cells and the envelope packaging of multiple nucleocapsids in symbiotic wasp calyx cells have not yet been established. Here, we evaluated envelope fusion during MbBV virion maturation by analyzing the ultrastructure of calyx cells using transmission electron microscopy (TEM). Thin-section TEM allowed us to obtain key information during envelope development in the nuclei of wasp calyx cells. Ovary development in the parasitoid wasp, *Chelonus inanitus*, is divided into six stages based on morphological changes [11], with the calyx developing at different stages in addition to exhibiting nuclear changes during virion production [5].

MbBV particles contain multi-nucleocapsids that have a diameter of 34–69.9 nm and a length of 13–126 nm with a discernable cap structure in two distal regions and a distinctive helix-tail-like structure that can be observed using cryo-electron microscopy [12]. In this study, we confirmed that a fused envelope containing multiple capsids occurs before MbBV virions are released. This study provides insights into the process underlying envelope-fusion-syncytium formation, wherein multiple nucleocapsids are packaged within a single envelope. Our results provide a basis for further investigation of the molecular mechanisms of the formation of viral fused envelopes during maturation.

## 2. Materials and Methods

### 2.1. Isolation of Insect Ovaries

Here, we used the protocol described in a published study [5] to isolate the insect ovaries. Fresh female adult *M. bicoloratus* ovaries were excised under a binocular stereomicroscope, placed in a 1.5 mL Eppendorf tube, and fixed in 2.5% glutaraldehyde in 1 × PBS (pH 7.4) at 4 °C for TEM.

### 2.2. Histological Staining

For histological examination, ovaries were fixed in 4% paraformaldehyde (PFA), embedded in paraffin, and sectioned at a thickness of 4–10 µm. Hematoxylin staining was performed on paraffin sections according to the standard protocol.

### 2.3. Sample Preparation for TEM Analysis

*M. bicoloratus* ovaries were fixed overnight at 4 °C with 2.5% glutaraldehyde in 1 × PBS (pH 7.4) in order to examine the virion ultrastructure. The fixed ovaries were rinsed three times with PBS and then fixed with 1% OsO_4_ for 1 h on ice. The samples were rinsed with distilled water three times, electron stained with 2% uranyl acetate, and rinsed three times with distilled water. The ovaries were dehydrated by sequential incubation in an ethanol series (30%, 50%, 70%, 80%, 90%, and 100% on ice, as well as 100% at 25 °C, 10 min each). To evaluate the ovary morphology, 200 nm sections were cut on an ultramicrotome (EM UC7, Leica Biosystems, Wetzlar, Germany) with glass knives, stained with tolonium chloride, and viewed under a light microscope (BX50, Olympus, Tokyo, Japan).

### 2.4. Ultramicrotomy and Electron Microscopy

The polymerized resin was sectioned using an ultramicrotome. Ultrathin sections (50 nm) were collected in a copper slot and subjected to post staining with lead citrate and uranyl acetate. After staining, the copper slot with the sample was used for TEM imaging. The sections were observed using a Hitachi HT7800 TEM (Tokyo, Japan) operated at 80 kV.

### 2.5. Cross-Sectional Area of Calyx Cell Nucleus and Fused Virus Envelopes

Calyx cell nucleus: The cross-sectional areas of the calyx cell nucleus at four stages, pre-VS, VS, assembly, and fusion, were determined using TEM, and then cell images were collected at low magnification. Cell nuclear areas were determined according to the nuclear membranes by Fiji ImageJ 2.1.0/1.53c for Win/Mac. One to six cells with a maximum cross-sectional area were used for determining calyx cell nucleus data from each preparation. A total of 45 cells were used for these measurements and were obtained from viewing triplicate preparations of wasps. 

Fused virus envelopes: To determine the cross-sectional area and envelope morphology of the virus, we defined viruses with only one envelope as containing one nucleocapsid; further, viruses possessing a high electron density were understood as unit viruses. Viruses with an envelope containing multiple nucleocapsids, and with a lower electron density, were defined as fused viruses. Due to the characteristics of ultrathin sections, the viruses whose nucleocapsids could not be identified in the envelope were not counted. The fused virus area was determined according to a single envelope using Fiji ImageJ 2.1.0/1.53c. A total of 30 to 35 fused viruses and unit viruses, respectively, at the fusion stage were individually used for the purposes of acquiring the virus cross-sectional area data from each preparation. All 100 of these fused viruses and unit viruses obtained from the measurements were then accumulated from the viewing of triplicate preparations. 

Envelope-fusion-syncytium formation types: Three types were classified based on the shapes in a single photo, and ten photos were used for determining the types from each preparation. All the data were obtained using triplicate preparations. 

### 2.6. Statistical Analysis

Data were analyzed using GraphPad Prism version 9 (GraphPad, Inc., La Jolla, CA, USA) and statistical significance was determined using Student’s *t*-test for unpaired experiments (two-tailed). Statistical significance was set at *p <* 0.05. The resulting data are presented as the mean ± standard error (SEM) of at least three independent experiments.

## 3. Results

### 3.1. Microplitis Bicoloratus Bracovirus Envelope Synthesis and Fusion Process

**Figure 1:**
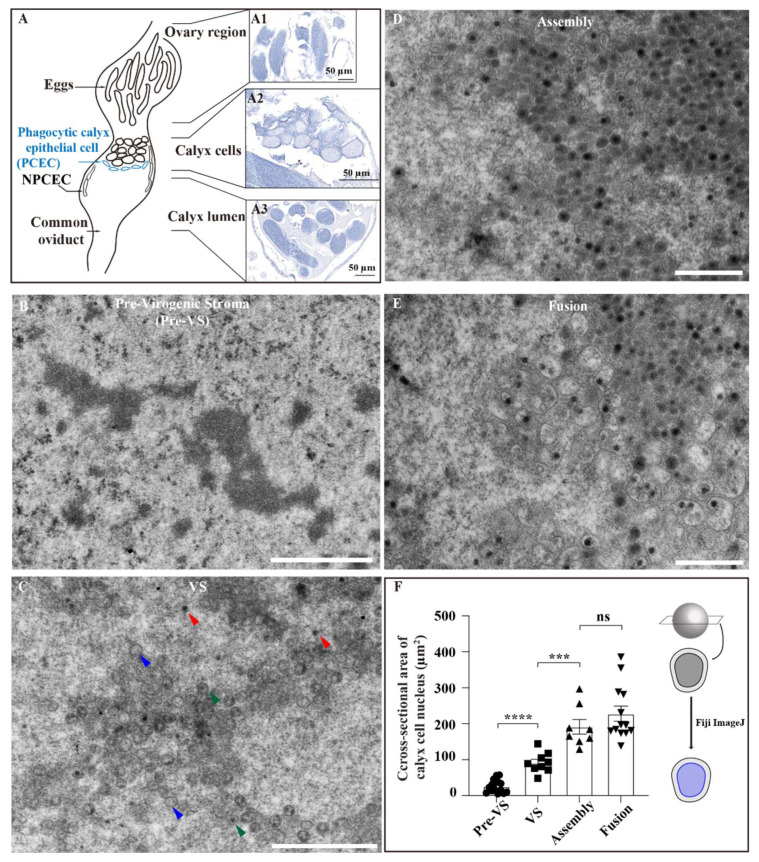
*Microplitis bicoloratus bracovirus* envelope synthesis and fusion process. (**A**) Schematic diagram and paraffin section of ovaries. Eggs exist in the ovary region. Phagocytic calyx epithelial cells (PCECs), non-phagocytic calyx epithelial cells (NPCECs), and calyx cells separate the ovary region from the calyx lumen. (**A1**) Ovary region. (**A2**) Calyx cells. (**A3**) Calyx lumen. Scale bar = 50 μm. (**B**) Pre-VS stage of MbBV. VS are visible. Scale bar = 1 μm. (**C**) VS stage. Envelopes synthesized de novo. Numerous envelopes and few nucleocapsids are synthesized. Envelopes do not cover free nucleocapsids. Blue arrowhead: empty envelope; red arrowhead: unpackaged nucleocapsids; green arrowhead: packaged virus particles. Scale bar = 1 μm. (**D**) Assembly stage. Nucleocapsids and envelopes complete one-to-one packaging. Scale bar = 500 nm. (**E**) Fusion stage. Early stage virus fusion. Fused envelopes can be found at the VS edge. Scale bar = 500 nm. (**F**) Cross-sectional area of the calyx cell nucleus in the four MbBV maturation stages. Each symbol represents the nuclear area of a single nucleus. *** *p* < 0.001; **** *p* < 0.0001; ns, no significant difference. Error bars represent the SEM. Unpaired Student’s *t*-test with the Holm–Sidak method was used for multiple comparisons *t*-test; *n* = 3. surface. Thus, calyx cells may be synchronized in relation to virus production; however, this requires further investigation. Next, we identified four stages of MbBV maturation using TEM: pre-virogenic stroma (pre-VS), VS, assembly, and fusion stages.

The ultrastructure of bracovirus has been reported previously [5,13,14,15]; however, the detailed processes of fused viral envelopes remains unclear. Electron microscopy is useful for evaluating transient virus changes. The ovary development of *C. inanitus* can be divided into six stages [5]; in this study, we focused on the sixth stage, in which the ovary contains mature virions and numerous calyx cells (Figure 1). To determine the location of calyx cells, we established paraffin sections, and then identified the egg region and four stages of virus maturation in distinct regions of the virus-producing calyx cells (Figure 1(A2)). These were located between the eggs (Figure 1(A1)) and the oviduct/calyx lumen (Figure 1(A3)). Calyx cells, which are tightly arranged, are separated from the ovary lumen by epithelial cells with an absence of virogenic stroma (VS); these are referred to as phagocytic calyx epithelial cells (PCECs), which play a role in phagocytosis [5]. Calyx cells appeared in different sizes, and the basal surface of the calyx cells was larger than the apical. 

Bracoviral envelopes are formed de novo in the nucleus [5,14]. Here, VS were widely present in the nuclei of calyx cells, where MbBV envelopes and nucleocapsids are thought to be synthesized. During the pre-VS stage, the calyx cells were small and contained VS without any fused viral envelope structure (Figure 1B). Therefore, in the VS stage, empty envelopes (blue arrowhead), unpackaged nucleocapsids (red arrowhead), and initially packaged virus particles were observed (green arrowhead, Figure 1C). Subsequently, numerous envelopes and unpackaged nucleocapsids continued to undergo assembly and were packaged one to one to form unit viruses in the assembly stage (Figure 1D). Although the unit viruses were in close contact with each other, they showed no tendency to fuse. As the unit viruses matured, fused viruses (in which one envelope assembled multiple nucleocapsids) appeared around the unit virus (Figure 1E). We measured the cross-sectional area of the calyx cell nucleus during the four phases of virus maturation (Figure 1F). The nucleus cross-sectional area significantly expanded compared with those in the previous stages. These changes in the cross-sectional area provided basic data for confirming that calyx cell development has important functions that contribute to the virus maturation process. These results demonstrate that the envelope was synthesized de novo and that the fusion occurred during the last stage of MbBV maturation.

### 3.2. Form of the Envelope-Fusion-Syncytium

**Figure 2:**
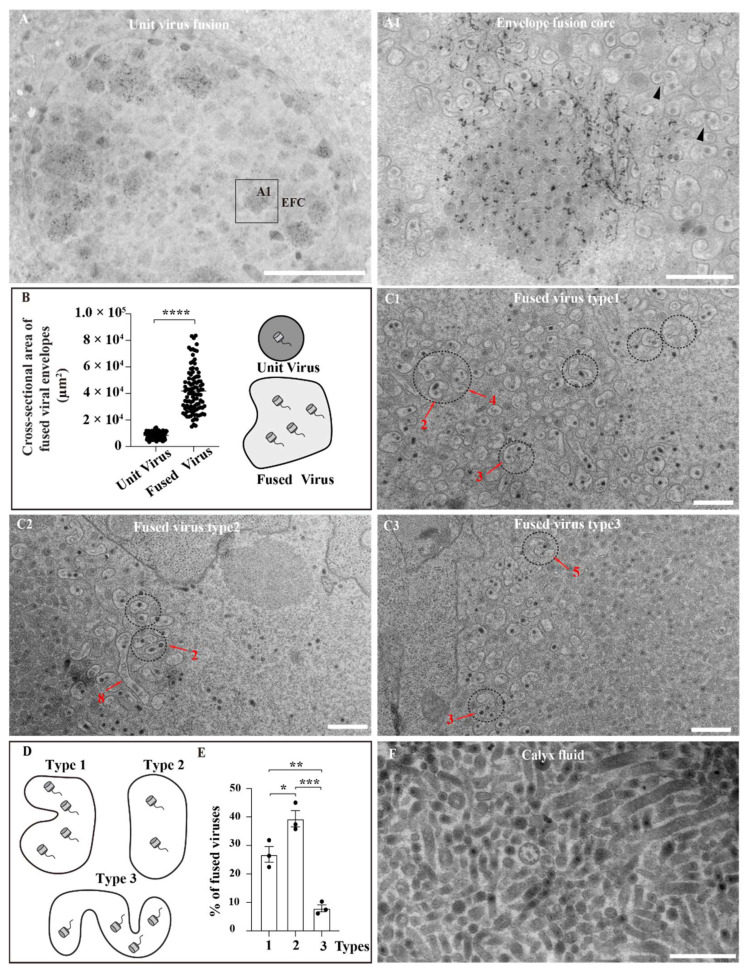
Envelope-fusion-syncytium formation. (**A**) Fused envelope mediated by organized structures. Numerous EFCs are evenly distributed in the calyx cell nucleus. Scale bar = 5 μm; (**A1**) EFC. The peripheral EFC is the active fused virus area. Most viruses in the central area are independent. Scale bar = 500 nm. (**B**) Unit virus and fused viral envelope cross-sectional area. **** *p* < 0.0001, error bars represent the SEM. Each symbol represents the nuclear area of a single viral envelope. Unpaired Student’s *t*-test with the Holm–Sidak method were used for multiple comparisons; *n* = 3. (**C1**) Fused virus type 1 and nucleocapsids (red arrow and number). Scale bar = 500 nm. (**C2**) Fused virus type 2 and nucleocapsids (red arrow and number). Scale bar = 500 nm. (**C3**) Fused virus type 3 and nucleocapsids (red arrow and number). Scale bar = 500 nm. (**D**) Schematic illustration of the envelope-fusion-syncytium formation types. (**E**) Frequency distribution of the three main fused virus structures. * *p* < 0.05, ** *p* < 0.01, *** *p* < 0.001. Error bars represent the SEM. Unpaired Student’s *t*-test with the Holm–Sidak method was used for multiple comparison; *n* = 3. (**F**) The mature MbBV accumulates in the ovary lumen. Scale bar = 500 nm.

The structure dominating the fused envelope was named the “envelope fusion core” (EFC) (Figure 2A), which was distributed in the nucleus as spheres. The EFCs naturally disappeared after fusion; light-colored virus clusters were fused viruses, whereas dark-colored clusters were viruses undergoing envelope fusion (unit viruses) (Figure 2(A1)). 

Viruses were produced in the VS and the envelope was fused near the EFCs. We measured the cross-sectional areas of fused viruses and unit viruses around the EFCs (Figure 2B). The cross-sectional area of fused viruses was significantly increased compared to that of unit viruses. The reason for the increase in the cross-sectional area contained by the envelope is unclear; however, the morphology of the fused virus envelope can be identified. We summarized distinguishable types of fused viruses (Figure 2(C1–C3)) and counted their frequency (Figure 2D,E). Notably, due to the limited resolution of electron microscopy, the classification of some fused viruses was difficult. Three fused types were observed during the formation of the envelope-fusion-syncytium: type 1 (26.9%), type 2 (39.4%), and type 3 (7.5%); the frequency of type 2 was the highest, followed by type 1 (Figure 2D,E). The commonality of these fusion structures is that of a single envelope containing multiple nucleocapsids. In addition, the number of nucleocapsids in the fused viruses was not constant (Figure 2(C1–C3)), red arrow and number). Our TEM images provide the first evidence of fused viral envelopes, indicating that envelope fusion in the nuclei of calyx cells is similar to membrane fusion during beta herpesvirus-mediated syncytium formation [6]. 

A previous study demonstrated that in calyx fluid, virions containing multiple nucleocapsids in a single envelope are highly concentrated and widely distributed in the calyx lumen [12]; in this study, no fused envelope was observed in the calyx lumen (Figure 2F). These data suggested that the envelope-fusion-yielding syncytia, with multiple nucleocapsids, only occurs during the fusion stage of the calyx cell nucleus and that various stabilized viruses are formed, which are then released into the ovary lumen.

### 3.3. PCECs Clear Non-Enveloped Nucleocapsids

**Figure 3:**
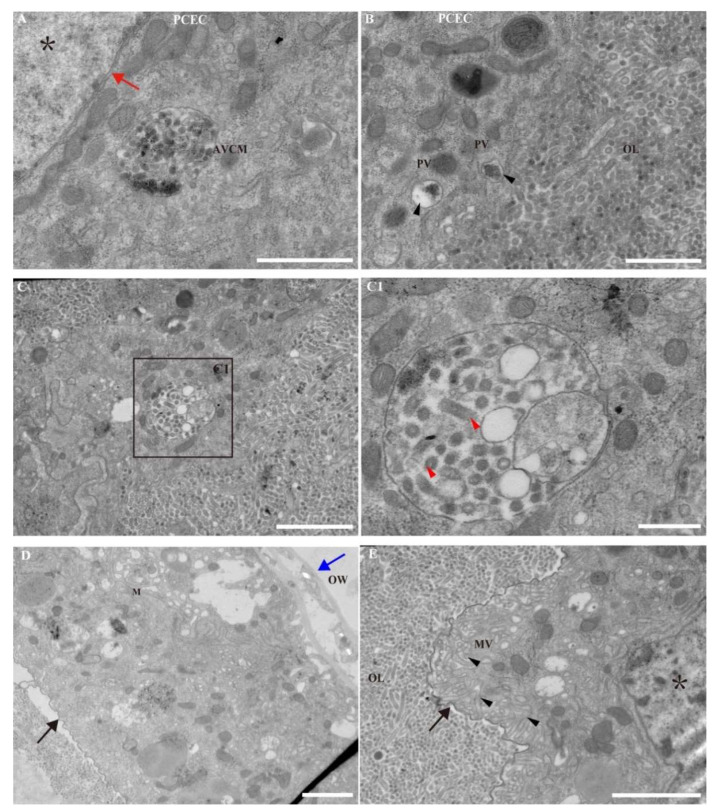
Phagocytic calyx epithelial cells (PCECs) clear non-enveloped nucleocapsids. (**A**) PCEC nucleus and autophagic vacuole containing MbBV (AVCM). Black asterisk: nucleus of PCECs. Red arrow: nuclear membrane. Scale bar in (**A**), 1 μm. (**B**) Phagocytic vacuole (PV) and ovary lumen (OL). Scale bar in (**B**), 1 μm. (**C**) AVCM and OL. Scale bar in (**C**), 2 μm; (**C1**) Non-enveloped nucleocapsid in AVCM. Scale bar in (**C1**), 500 nm. (**D**) Non-phagocytic ovary epithelial cells with a cuticular line and ovary wall (OW). Black arrow: cuticular lining; blue arrow: OW; Scale bar in (**D**), 2 μm. (**E**) Non-phagocytic ovary epithelial cells microvilli and OL; black arrow: cuticular line; black asterisk: nucleus of non-phagocytic ovary epithelial cells; OL: ovary lumen; MV: microvilli; scale bar in (**E**), 2 μm.

Interestingly, we observed some nucleocapsids that were not assembled into envelopes. They should have been released into the calyx lumen via lysis of the calyx cells; however, we did not find non-enveloped nucleocapsids in the calyx lumen (Figure 2F). Further, the cuticular line of epithelial cells separates the ovary lumen and ovary wall [14]. Here, we redesignated these two types of epithelial cells (i.e., PCECs and NPCECs; Figure 1A). PCECs, in the lower calyx region, separated calyx cells and calyx fluid in the calyx lumen (Figure 3A–C). In contrast, the NPCECs were found with microvilli and a cuticular line, which separated the ovary wall and lumen (Figure 3D,E).

An autophagic vacuole containing MbBV (AVCM) was identified near the PCEC nucleus (red arrow) (Figure 3A), and phagocytic vacuoles were detected on the border of the ovary lumen and the PCECs (Figure 3B), suggesting that PCECs clear debris from lysed calyx cells. Viral nucleocapsids were identified in the autophagic vacuole of PCECs (Figure 3C). Importantly, obtaining evidence that non-enveloped nucleocapsids in the PCECs were phagocytosed due to the absence of an envelope—rather than being enveloped nucleocapsids that were phagocytosed with a fusion of their envelope into the phagocytic vacuolar membrane—is an interesting area for further research. These results suggest that PCECs may clear the debris that is released by calyx cells, which includes non-enveloped nucleocapsids. These results imply that envelope-fusion-multiple-nucleocapsid-syncytium may contain a marker of mature particle formation in order to avoid phagocytosis by PCECs, and to protect mature bracovirus virions.

## 4. Discussion

Our ultrastructure analysis highlights the importance of envelope-fusion-syncytium formation of specific structures for viral maturation. These results agree with those of previous studies showing that envelopes are formed de novo [5,14]; furthermore, that the envelope packages of nucleocapsids develop one to one in the assembly stage [5]. We identified EFCs, in which the fusion stage produces three types of multi-cored viral particles. The enveloped and matured virions are released into the calyx lumen by lysing calyx cells that cross the epithelial layer [5,14,16]. We redesignated two types of epithelial cells on the border of the ovary lumen, i.e., PCECs and NPCECs. Furthermore, we determined the details of fused envelopes, providing evidence that poly-nucleocapsids in an envelope are formed by envelope-fusion-syncytium formation.

We identified the EFC, which is a specific structure in the nucleus at the calyx fusion stage, suggesting that a fused envelope is required for bracovirus virion maturation. We divided MbBV maturation into four stages: pre-VS, VS, assembly, and fusion. EFCs were found during the last stage of virus maturation, which was redesigned as a fusion stage (Figure 1E). We explored the morphology of MbBV envelopes and found that calyx cells exhibited organized behavior for efficient virus production, including calyx cell expansion (Figure 1F), syncytium types (Figure 2D), and debris phagocytosis (Figure 3C).

Recently, an envelope protein, MdBVe46, from *Microplitis demolitor bracovirus* was shown to be necessary but not alone sufficient for virus maturation [16]. This raised the question whether the fused envelope is regulated by calyx cells. Understanding the mechanism of formation of fused viral envelopes in calyx cells would be valuable for future studies.

Envelope-fusion-syncytium formation is a hypothesis as to how one envelope containing multiple nucleocapsids can be formed (Figure 2B). After the virus assembly is completed, the unit viruses on the edges of EFCs have space in the stromata to bring viruses together. Envelope fusion is a dynamic process; further, envelope fusion and cell syncytium formation possess many similarities, and both are mediated by viral proteins, forming a state of multiple nuclei or multiple nucleocapsids. Although some studies suggest that envelope proteins affecting cell syncytium formation also affect virus maturation [6,17,18,19], the relationship of these proteins with envelope fusion is unclear. Since envelope fusion depends on envelope proteins, diversity exists in the fusion structure. In addition, the varying number of nucleocapsids further demonstrates the complexity of the fusion mechanism.

Our results suggest that envelope fusion-derived debris of calyx cells and non-enveloped nucleocapsids are cleared by phagocytic calyx cells (Figure 3). BVs release the virus into the calyx lumen by lysing calyx cells after fused envelope formation [15,20,21,22]. A thin epithelial cell layer that forms the border between calyx cells and the ovary lumen stores the mature virions, and epithelial cells perform phagocytosis to clear debris [5]. We identified two types of epithelial cells in different locations: the first are those separating the calyx cells and lumen (Figure 3A), in which autophagic vacuole containing MbBVs and phagocytic vacuoles were found; the second are those separating the ovary wall and lumen with two specific structures, i.e., the microvilli and cuticle (Figure 1A and Figure 3D). We redesignated the former as PCECs, and the latter as NPCECs. These results identify specific epithelial cells for further phagocytotic research.

In summary, our data suggests that the formation of a fused envelope is a key process during MbBV maturation. Our data also fills a key knowledge gap in the understanding of envelope development. The mechanism of envelope fusion needs to be explored further through molecular biology experiments.
